# Oral Mucositis Induced By Anticancer Therapies

**DOI:** 10.1007/s40496-015-0069-4

**Published:** 2015-10-19

**Authors:** Sali Al-Ansari, Judith A. E. M. Zecha, Andrei Barasch, Jan de Lange, Fred R. Rozema, Judith E. Raber-Durlacher

**Affiliations:** Department of Medical Dental Interaction, Academic Center for Dentistry Amsterdam, University of Amsterdam and VU University, Gustav Mahlerlaan 3004, 1081 LA Amsterdam, The Netherlands; Department of Oral- and Maxillofacial Surgery, Academic Medical Center, University of Amsterdam, Meibergdreef 9, 1105 AZ Amsterdam, The Netherlands; Department of Surgery, Weil Cornell Medical College, 528 E 68th Street, New York, NY 10065 USA; Department of Periodontology, Academic Center for Dentistry Amsterdam, University of Amsterdam and VU University, Gustav Mahlerlaan 3004, 1081 LA Amsterdam, The Netherlands

**Keywords:** Oral mucositis, Stomatitis, Cytotoxic cancer therapy, Chemotherapy, Radiation therapy, Targeted therapies, Mucositis management

## Abstract

Oral mucositis induced by conventional cytotoxic cancer therapies is a common and significant clinical problem in oncology. Mucositis symptoms, which include severe pain, may lead to dose reductions and unplanned interruptions of chemotherapy and/or radiotherapy, and often affect patients' quality of life. In addition, ulcerative mucositis represents a risk factor for local or systemic infectious complications that may be life-threatening in immunosuppressed patients. The development of biologically based targeted cancer therapies, which aim to block the growth, spread, and survival of tumors by interfering with specific molecular targets, may have reduced mucosal injury, but did not eliminate it. This article will review the epidemiology, pathobiology, and management of oral mucositis associated with conventional cytotoxic therapies for malignant diseases and will briefly summarize emerging information on oral mucosal injury associated with targeted therapies. Considerations for future research aimed at the development of more efficient and effective supportive care approaches will be presented, with emphasis on the contribution of dental researchers and clinicians in these efforts.

## Introduction

Oral mucositis (OM), an inflammatory condition of the oral and oropharyngeal mucosa induced by cytotoxic chemotherapy and/or radiotherapy, represents a major clinical problem in oncology. In its most severe form, OM presents as confluent, deep ulcerations. Pain associated with mucositis often impairs a patient’s functional status and quality of life [1–3]. In patients treated with chemotherapy alone or combined chemoradiation regimens, the whole gastrointestinal tract may be affected. Weight loss is common, and patients may require gastrostomy or parenteral feeding. Severe OM may lead to dose reductions and unplanned interruptions of cancer therapies. In addition, ulcerative mucositis, characterized by disruption of the integrity of the epithelial barrier, represents a significant risk factor for systemic infectious complications, particularly in neutropenic or otherwise immunocompromised patients. These clinical consequences are associated with increased use of healthcare resources and may negatively impact survival [4].

Despite major progress in our understanding of the pathobiology of chemotherapy and/or radiotherapy-induced mucositis, and the availability of interventions in selected patient populations, OM and its associated symptoms still represent an unmet need [5•].

Recently, major advances have been achieved in the field of biologically based therapies for cancer. These novel “targeted” approaches aim to block the growth, spread, and survival of tumors by interfering with specific molecular targets, and include hormone receptor-blocking therapies, signal transduction inhibitors, gene expression modulators, apoptosis inducers, angiogenesis inhibitors, immune response enhancers, and toxin delivery molecules [6]. A substantial number of targeted drugs have been approved by regulatory authorities for the treatment of a variety of malignancies, and can be used alone or in combination with conventional cytotoxic anticancer therapies [7••].

As expected, targeted cancer therapies are generally less toxic to normal cells than traditional chemotherapy drugs. However, it has become increasingly clear that targeted cancer therapies can have substantial side effects, including effects on oral tissues [8]. The clinical features of oral lesions induced by some of these new therapies differ from those of conventional OM, and the current understanding of their pathobiological pathways is limited.

This narrative review paper will focus on OM associated with conventional malignant disease therapies, and we will briefly summarize emerging information on oral lesions associated with targeted therapies. In addition, we will present considerations for future research aimed at developing more efficient and effective supportive care approaches, with emphasis on the contribution of dental researchers and clinicians in these efforts.

## Oral Mucositis Among Different Types of Cytotoxic Treatment

Prevalence and incidence data on OM vary considerably. Reasons for inconsistencies include a heterogeneity of standardized scoring criteria, variation among tumor type locations, and different treatment regimens [9••]. In addition, there may be genetic and ethnic differences among patients in studies performed in different parts of the world. Moreover, most data are derived from clinical trials in which OM was not a primary study endpoint, and therefore its frequency is likely underreported. On the other hand, OM can be easily confused with infections or other oral mucosal conditions, which may have also affected figures reported in the literature.

### Oral Mucositis Associated with Hematopoietic Stem Cell Transplantation

Oral mucositis (any grade) develops in approximately 75 % to 100 % of patients undergoing myeloablative autologous (with the patient’s own stem cells) or allogeneic (with donor-derived stem cells) hematopoietic stem cell transplantation (HSCT) [10]. Myeloablative conditioning regimens cause irreversible pancytopenia, and stem cell support is required to rescue marrow function in order to prevent aplasia-related death [11]. Prospective studies have reported that conditioning regimens containing high-dose melphalan, busulfan, and/or cyclophosphamide, with or without total body irradiation (TBI), were associated with severe OM [12, 13]. The incidence of severe OM (WHO grades 3–4) may be lower for individuals treated with conditioning protocols without TBI [14••]. A large prospective European study found that 67 % of multiple myeloma patients treated with high-dose melphalan followed by autologous HSCT developed ulcerative OM (WHO grade ≥ 2), whereas ulcerative OM occurred in 60 % of non-Hodgkin’s lymphoma patients treated with carmustine, etoposide, cytarabine, melphalan (BEAM) chemotherapy, and autologous HSCT [15].

Over the last two decades, less toxic conditioning regimens have been introduced, based on observations that alloreactivity of transplanted donor immunocompetent cells against host tumor cells (graft-versus-tumor/leukemia effect) plays a major role in eradicating malignancies, thus expanding the availability of HSCT to older patients and those with comorbidities. Non-myeloablative (NMA) conditioning protocols are the mildest form, typically causing minimal pancytopenia but considerable immunosuppression, allowing full engraftment of donor cells. Reduced-intensity conditioning regimens (RIC) form an intermediate category of therapy. RIC regimens differ from myeloablative conditioning in that the dose of chemotherapy agents and/or TBI is reduced by at least 30 %. Both NMA and RIC regimens are associated with reduced incidence and severity of OM [16, 17]. However, allogeneic transplant patients thus treated require graft-versus-host disease prophylaxis, which may increase the risk of OM. There are relatively scant data on the incidence and severity of OM associated with NMA and RIC.

### Chemotherapy-Induced Oral Mucositis

The frequency of chemotherapy-induced OM in patients with solid tumors is not well documented, and varies significantly among various studies [18, 19]. One explanation for the divergent findings may be that most studies follow patients only during their first cycle of chemotherapy, whereas the incidence of mucositis may increase significantly in subsequent cycles due to cumulative effects. For example, among common chemotherapy regimens in breast cancer, ulcerative OM was reported in about 20 % of patients during the first cycle of treatment. If these patients received the same dose of the same drugs in a second cycle, the frequency of OM increased to 70 % [20]. In patients undergoing chemotherapy treatment regimens for lymphoma or solid tumors, 20 % to 40 % developed any grade of OM [21–23]. A systematic review evaluating multiple studies reported that standard chemotherapy regimens for non-Hodgkin’s lymphoma occasionally resulted in severe OM (3–10 %), and similar rates were observed in breast cancer patients treated with doxorubicin- and taxane-based regimens [18]. In addition, patients with lung cancer who received platinum-based chemotherapy and patients with advanced colorectal cancers treated with standard regimens containing 5-fluorouracil were at low risk for severe OM. In a recent prospective study involving 298 patients treated with myelosuppressive chemotherapy for solid tumors, 120 patients (40.3 %) developed WHO grade 1 OM, 15 patients (5 %) showed WHO grade 2, and only 3 patients (1 %) had severe OM (WHO grades 3–4) [24•]. It should be noted, however, that even mild mucositis may represent a burden to patients.

### Radiotherapy-Induced Oral Mucositis

In patients undergoing radiation therapy for head and neck cancer (HNC), OM is a frequent toxicity, affecting almost all patients in which areas of the oral or oropharyngeal mucosa are included in the treatment field. Sutherland and colleagues reported that approximately 60 % of patients receiving standard radiotherapy developed severe OM [25]. In advanced HNC (about 60 % of clinical presentations), combined chemoradiation has been associated with improved locoregional disease control and organ preservation, and has become an accepted standard of care for tumors that cannot be removed surgically, or for cases in which surgery causes unacceptable changes to speech or swallowing [26–28]. However, this is at the cost of greater toxicity, including severe OM.

In a systematic review of 33 studies involving 6181 patients, in which the incidence of OM was investigated in patients treated with different radiation therapy modalities as well as chemoradiation, the mean incidence of OM was found to be 80 % [29]. Over one-half of patients (57 %) who received altered fractionation radiotherapy experienced severe OM, compared to 34 % of patients who received conventional radiotherapy and 43 % of patients treated with combined chemoradiation protocols. Rates of hospitalization due to OM reported in three studies were 16 % overall and 32 % for patients treated with altered fractionated protocols. Eleven percent of patients had OM sufficiently severe to interrupt radiation therapy.

Vera-Llonch and colleagues conducted a retrospective study to evaluate the incidence and clinical impact of OM in 450 patients receiving radiotherapy for HNC [30], reporting the occurrence of OM in 83 % of patients, among which 29 % of cases were severe. Severe OM was associated with treatment breaks and hospitalization, and was more likely to occur in patients with nasopharyngeal carcinoma or oropharyngeal tumors who received concomitant chemotherapy. Elting and coworkers reported virtually identical incidence of OM in patients with oral cavity or oropharynx tumors (99 % overall; 85 % grade 3–4) and those with tumors of the larynx or hypopharynx (98 % overall; 77 % grade 3–4). In this prospective multicenter study, patients received a cumulative radiation dose of least 40 Gray (Gy) in single daily fractions, with or without subsequent boost and/or chemotherapy [3].

Conventional 2D and 3D radiotherapy uses large fields and a series of field reductions to provide sequentially higher doses to the primary tumor. Intensity-modulated radiotherapy (IMRT) has emerged as an effective technique for delivering the full radiation dose to the tumor and regions at risk while reducing exposure to healthy tissues. The effect of IMRT was examined in 160 HNC patients to determine whether it could reduce the incidence and/or severity of OM and consequent dose delays and reductions [31]. Patients were treated with standard radiotherapy or IMRT, with or without chemotherapy. Mucositis occurred in 97–100 % of patients, among which ≥ 69 % developed severe OM. Although there was a trend toward decreased incidence of severe OM in patients who received IMRT compared with standard radiotherapy, there was no significant difference in dose delays or reductions. Another study of 158 HNC patients reported that more patients treated with conventional radiotherapy exhibited OM compared with those treated with IMRT (46.5 % versus 16.9 %) [32].

The addition of epidermal growth factor receptor (EGFR)-targeted therapies to radiotherapy regimens has improved treatment outcomes [33]. Combined treatment with cetuximab and IMRT was found to be more likely to cause acute adverse events, including OM, in patients with nasopharyngeal carcinoma than protocols with induction chemotherapy followed by concomitant cisplatin and IMRT [34].

## Risk Factors

There are still many unanswered questions about the risk factors for developing OM, but historically, risk factors have been attributed to both therapy and patient characteristics [35]. As mentioned above, treatment variables that may affect the incidence and the severity of OM include the type, dose, and schedule of systemic cytotoxic drugs delivered, radiation dose and field, and concomitant use of chemotherapy and radiation. Studies have shown that the risk of OM increases as the intensity of therapy increases [36].

Patient-related risk factors are more complex and, for the most part, are poorly defined. Despite similarities in diagnosis and treatment, patients are not at equal risk of developing mucositis. Among patient-associated factors, age, malnutrition, gender, pre-existing medical conditions, alterations in salivary production and composition, poor oral health, and mucosal trauma have been reported to influence the risk of OM (reviewed in [37]). Poor dental health, particularly periodontal disease, has been identified as an environmental factor that may increase the severity of OM (discussed in more detail below) [24•, 38]. Reducing oral bacterial load and periodontal inflammation was associated with a lower prevalence of OM in HSCT recipients [39–41]. There has been increased interest recently in the role of the oral microbiome in OM risk [42, 43•, 44••], with studies suggesting that shifts in the composition of the oral microbiome during chemotherapy influence OM severity [45]. *Porphyromonas gingivalis* and other periodontal pathogens have been identified as explanatory variables for oral ulcerations [46]. In addition, fungi and viruses that are typically associated with mucosal injury have been studied for their potential involvement in the development of ulcerative OM, but no firm conclusions can be drawn [46–50].

Genomic differences, which are major determinants of toxicity risk, have been identified among patients with head and neck cancer who received radiotherapy [20, 51–53]. Genetic determinants of chemotherapy-induced risk of mucositis include genes that regulate the availability of active drug metabolites. For example, evaluation of genetic variations in folate-metabolising enzymes may help in identifying patients at greater risk for methotrexate toxicity, although enzyme deficiencies may be relatively rare. In contrast, differences in the expression of genes associated with biological pathways that drive mucositis are more common. For instance, genetic polymorphisms associated with the expression of inflammatory mediators such as TNF-α have been implicated in OM risk [54]. The tumor itself has recently become appreciated as playing a role in OM risk [20, 55]. The inflammatory response induced by the tumour, together with inflammation from treatment-induced cytolysis, may contribute to adverse events, including OM [56••].

## Pathobiology

The cytotoxic effects of antineoplastic therapies are not limited to tumor cells, but also affect normal tissues. Historically, OM was viewed exclusively as the result of nonspecific toxic effects of radiation and/or chemotherapy on rapidly proliferating basal epithelial cells, resulting in clonogenic cell death and consequently in tissue atrophy and ulceration. However, animal studies have indicated that the pathobiology of OM is much more complex, which prompted prompt Sonis to propose a model involving connective as well as epithelial tissues [57]. This five-phase model describes a cascade of interrelated and overlapping events. Phases include initiation, upregulation and activation, signal amplification, ulceration, and healing.

The initiation phase is characterized by radio- and/or chemotherapy-induced DNA and non-DNA damage that results in injury of basal epithelial, submucosal, and endothelial cells. These cells release endogenous damage-associated molecular patterns (DAMPs), which then bind to specific receptors and play an integral role in initiating inflammation toxicity [58]. In response to this damage, oxidative stress results in the formation of reactive oxygen species (ROS) inside injured cells. ROS further damage cell membranes, stimulate macrophages, and trigger molecules that activate transcription factors, including nuclear factor (NF)-$\kappa$B [59]. NF-$\kappa$B can be considered the “gatekeeper” for inflammatory pathways involved in mucositis. Its activation precedes peaks in proinflammatory cytokines such as tumor necrosis factor (TNF)-α, interleukin (IL)-6, and IL-1β, and upregulates cyclooxygenase-2 (COX-2) in submucosal fibroblasts and endothelial cells [60–62].

Many of the molecules induced by this primary response have the ability to alter the local tissue response through feedback loops. For example, TNF-α activation may generate positive feedback on NF-κB to amplify its response (signal amplification phase) and initiate mitogen-activated protein kinase (MAPK) signaling, leading to activation of c-Jun N-terminal kinase (JNK) signaling [63]. NF-$\kappa$B independent pathways such as the ceramide pathway also play a role, resulting in apoptosis of submucosal and basal epithelial cells, leading to mucosal ulceration (ulcerative phase). Recent studies suggest the involvement of deregulated expression of metalloproteinases (MMPs) in the pathobiology of mucositis [62, 64•].

The ulcerative phase comprises loss of mucosal integrity and microbiological colonization by oral bacteria. Bacterial cell wall products are capable of extending mucosal damage as they stimulate infiltrating macrophages to produce additional pro-inflammatory cytokines.

Healing of ulcerations is associated with epithelial proliferation, often concurrent with hematopoietic recovery, reestablishment of local microbial flora, and absence of factors that interfere with wound healing such as infection and mechanical irritation [65]. The extracellular matrix (ECM) is a complex structural network of fibrous proteins, proteoglycans, and glycoproteins that plays a role in signaling between tissues. ECM stimulates epithelial cell migration, proliferation, and differentiation, leading to renewal of the mucosa. [66].

## Presentation and Clinical Course of Oral Mucositis

**Fig. 1:**
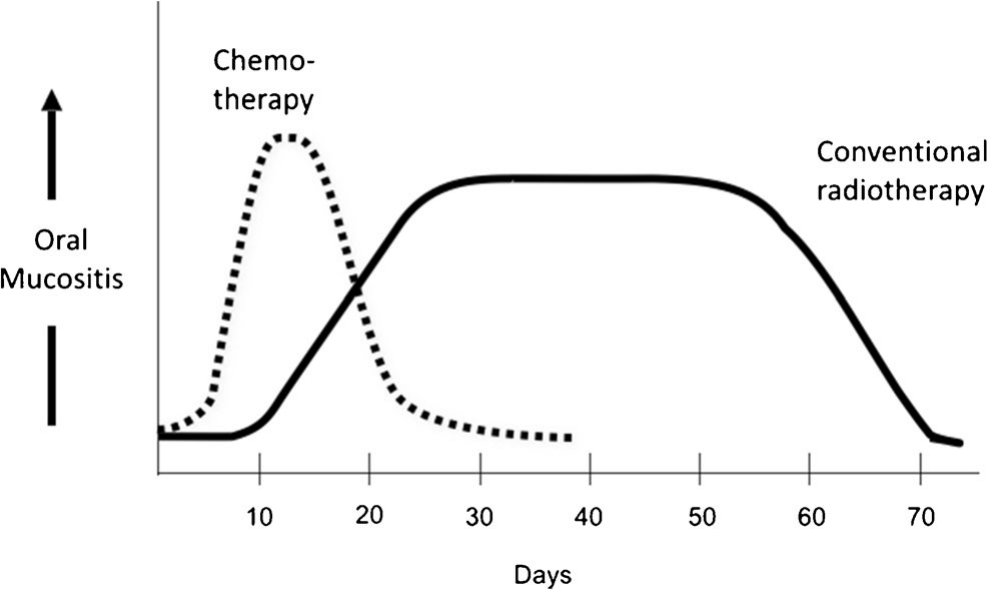
Clinical differences in onset, severity, and resolution of chemotherapy- and radiation-induced oral mucositis. In some patients treated with radiotherapy, oral mucositis may last for longer periods and may become chronic. Hyperfractionated radiotherapy, combined chemoradiation regimens, or radiotherapy combined with a targeted agent may lead to increased mucositis severity (not depicted)

While there is similarity in the cellular events of chemotherapy-induced and radiation-induced OM, there are differences in the kinetics of treatment, affecting the clinical course (Fig. 1). Among patients receiving cycled chemotherapy or conditioning regimens before HSCT, the first signs of OM usually begin about 3–4 days after drug infusion, and ulcer formation begins shortly thereafter. OM peaks about 7 to 14 days later, and then resolves within another 5–10 days.

Radiotherapy-induced OM has a more gradual clinical course, as radiotherapy is typically administered in small fractions with a total of about 10 Gy per week, and continues until a total dose of 60 to 70 Gy has been delivered. Clinical signs of OM generally appear at cumulative doses of about 15 Gy, and reach full severity at 30 Gy. Nowadays, patients may receive treatment involving chemoradiation regimens (usually with cisplatin or carboplatin) or radiotherapy with concurrent cetuximab, which are associated with increased OM severity [67–69]. Ulcerations typically resolve 2–4 weeks after the completion of therapy, but may last longer in some patients.

In both chemotherapy- and radiotherapy-induced OM, mucosal erythema is often the first manifestation, and it may be accompanied by feelings of burning. In patients who receive chemotherapy for the treatment of solid tumors, OM may not progress to severe forms. However, many patients develop one or more deep ulcerations that may be covered with a pseudomembrane. Their borders are generally poorly defined and do not display peripheral erythema. Ulcer development is associated with increased pain, since epithelial cell loss results in the exposure of the richly innervated underlying connective tissue. OM often impairs food intake.

OM lesions are most frequently found on the buccal and labial mucosa, lateral and ventral tongue, floor of the mouth, and the soft palate. The more heavily keratinized mucosal surfaces are usually spared by OM, but may be affected by viral and/or fungal diseases (*Herpesviridae* family, *candida* spp.).

## Stomatitis Associated with Targeted Therapies

Targeted anticancer agents influence or inhibit the signaling of many cellular targets, including mammalian target of rapamycin (mTOR), EGFR, vascular endothelial growth factor receptor (VEGFR), human epidermal growth factor receptor (EGF)-2, and several (multi-targeted) tyrosine kinases. Many monoclonal antibodies and small molecule inhibitors are now used to improve survival for a wide variety of malignancies.

Oral toxicities caused by these agents differ clinically, and likely also pathobiologically, from conventional OM. Therefore, the broader term “stomatitis” is preferred to “mucositis” for describing the mucosal injuries and other oral toxicities (e.g., mucosal sensitivity, taste alterations, dry mouth, gingival/jaw bone necrosis) associated with selected targeted agents [70–73]. Although oral mucosal lesions are usually mild and self-limiting, lesions may persist over long periods, presenting a significant burden to patients.

The prevalence of oral toxicities (any grade) has been reported at 38 % for sunitinib, 28 % for sorafenib, and 4 % for pazopanib in patients with renal cancer [71]. In meta-analyses conducted by Elting and coworkers, stomatitis was most frequently reported among patients treated with bevacizumab, erlotinib, sorafenib, or sunitinib [74].

mTOR inhibitors (everolimus and temsirolimus) are approved for the treatment of renal cell cancer and selected other malignancies. mTOR inhibitor-associated stomatitis (mIAS) resembles aphthous stomatitis, characterized as distinct ovoid ulcers with a central gray area surrounded by a ring of erythema [70]. These lesions typically present with a rapid onset (usually within 5 days), most frequently in the first cycle of therapy. Similar to conventional OM, mIAS almost exclusively affects the non-keratinized, movable oral surfaces. Even small ulcerations can cause significant pain, and mucosal sensitivity may occur in the absence of clinical changes. The use of assessment tools driven primarily by ulceration size may underestimate mIAS, and assessment should include patient-reported outcomes [75]. In a systematic review evaluating 44 studies, mIAS was identified as the most frequent adverse event overall (73.4 %), accounting for 27.3 % of dose reductions and 13.1 % of therapy discontinuation [76].

## Management Considerations

### Conventional Oral Mucositis

Mucositis management relies on symptom management and prevention of complications, which includes pain control, nutritional support, and prophylaxis/treatment of secondary infections [77]. Although these components continue to be of great importance, research has also identified a number of specific strategies to prevent the onset or reduce the severity of OM. Clinical practice guidelines have been prepared for the management of OM based on evidence and expert opinion [78–80]. The Multinational Association of Supportive Care in Cancer/International Society of Oral Oncology (MASCC/ISOO) recommends or suggests (depending on the level of supporting evidence) interventions for the prevention or treatment of OM in specific patient populations. Oral care is a key factor in the prevention and mitigation of oral injury; thus, reducing the microbial load and educating the patient regarding oral hygiene is very important. Other preventive measures include cryotherapy, keratinocyte growth factor-1, low-level laser therapy, benzydamine mouthwash, and zinc (Table 1).

**Table 1:** Multinational Association for Supportive Care in Cancer/International Society for Oral Oncology (MASCC/ISOO) Clinical Practice Guidelines for Oral Mucositis. Modified from [78]

Intervention/mode of administration	Purpose	Cancer treatment	Level of evidence
Recommendations IN FAVOR of an intervention (strong evidence supports effectiveness in the treatment setting listed):			
Oral cryotherapy for 30 minutes	Prevention of OM	Patients receiving bolus 5-fluorouracil chemotherapy	Level II
Recombinant human keratinocyte growth factor-1 (palifermin) at a dose of 60 μg/kg per day for 3 days prior to conditioning treatment and for 3 days after transplant	Prevention of OM	Patients receiving high-dose chemotherapy and TBI, followed by autologous stem cell transplantation, for a hematological malignancy	Level II
Low-level laser therapy (wavelength at 650 nm, power of 40 mW, and each square centimeter treated with the required time to a tissue energy dose of 2 J/cm^2^)	Prevention of OM	Patients receiving HSCT conditioned with high-dose chemotherapy, with or without TBI	Level II
Patient-controlled analgesia with morphine	Pain reduction	Patients undergoing HSCT	Level II
Benzydamine mouthwash	Prevention of OM	Patients with HNC receiving moderate dose radiation therapy (up to 50 Gy), without concomitant chemotherapy	Level II
Suggestions IN FAVOR of an intervention (weaker evidence supports effectiveness in the treatment setting listed):			
Oral care protocols	Prevention of OM	All age groups and across all cancer treatment modalities	Level III
Oral cryotherapy	Prevention of OM	Patients receiving high-dose melphalan, with or without TBI, as conditioning for HSCT	Level III
Low-level laser therapy (wavelength around 632.8 nm)	Prevention of OM	Patients undergoing radiotherapy, without concomitant chemotherapy, for HNC	Level III
Transdermal fentanyl	Pain reduction	Patients receiving conventional or high-dose chemotherapy, with or without TBI	Level III
2 % morphine mouthwash	Pain reduction	Patients receiving chemoradiation for HNC	Level III
0.5 % doxepin mouthwash	Pain reduction	All patients with OM-induced pain	Level IV
Systemic zinc supplements administered orally	Prevention of OM	HNC patients receiving radiation therapy or chemoradiation	Level III
Recommendations AGAINST interventions (strong evidence indicates lack of effectiveness in the treatment setting listed):			
PTA (polymyxin, tobramycin, amphotericin B) and BCoG (bacitracin, clotrimazole, gentamicin)	Prevention of OM	Patients receiving radiation therapy for HNC	Level II
Iseganan antimicrobial mouthwash	Prevention of OM	Patients receiving high-dose chemotherapy, with or without TBI, for HSCT or in patients receiving radiation therapy or concomitant chemoradiation for HNC	Level II
Iseganan antimicrobial mouthwash	Prevention of OM	Patients receiving high-dose chemotherapy, with or without TBI, for HSCT or in patients receiving radiation therapy or concomitant chemoradiation for HNC	Level II
Sucralfate mouthwash	Prevention of OM	Patients receiving chemotherapy for cancer (I), or inpatients receiving radiation therapy (I) or concomitant chemoradiation (II) for HNC	Level I, II
Sucralfate mouthwash	Treatment of OM	Patients receiving chemotherapy for cancer (I), or in patients receiving radiation therapy (II) for HNC	Level I, II
Intravenous glutamine	Prevention of OM	Patients receiving high-dose chemotherapy, with or without TBI, for HSCT	Level II
Suggestions AGAINST interventions (weaker evidence indicates lack of effectiveness in the treatment setting listed):			
Chlorhexidine mouthwash	Prevention of OM	Patients receiving radiation therapy for HNC	Level III
Granulocyte-macrophage-colony-stimulating factor mouthwash	Prevention of OM	Patients receiving high-dose chemotherapy, for autologous or allogeneic HSCT	Level II
Misoprostol mouthwash	Prevention of OM	Patients receiving radiation therapy for HNC	Level III
Systemic pentoxifylline, administered orally	Prevention of OM	Patients undergoing HSCT	Level III
Systemic pilocarpine, administered orally	Prevention of OM	Patients receiving radiation therapy for head and neck cancer (III), or patients receiving high-dose chemotherapy, with or without TBI, for HSCT (II)	Level II, III

*OM* oral mucositis, μg microgram, *kg* kilogram, *nm* nanometer, *mW* milliwatt, *J* Joule, *cm* centimeter, *Gy* Gray, *HSCT* hematopoietic stem cell transplantation, *TBI* total body irradiation, *HNC* head and neck cancer

### Stomatitis

There are no evidence-based guidelines for the management of mucosal lesions associated with targeted therapies. Management begins with assessment and oral hygiene measures, diet modifications, and pain management. In most cases, pain can be controlled with locally applied products containing lidocaine or doxepin and mucosal coating agents. In persistent cases, treatment with local or systemic corticosteroids can be considered [75]. Secondary candidiasis is a common side effect of topical steroid therapy. If this occurs, topical antifungal therapy should be initiated. However, systemically absorbed azole antifungal agents may increase the toxicity of mTOR inhibitors.

## Future Research Directions

Progress in mucositis research largely relies on investigations aimed at identifying potential targets for preventive and therapeutic interventions. A number of agents are at various stages of clinical development, and studies are underway on novel delivery mechanisms and risk prediction models that can facilitate the selective use of interventions in a cost-effective manner [5•]. Here we will discuss some potential lines of approach in which cross-pollination between recent concepts in oral/periodontal medicine and mucositis research may lead to new insights and points of departure for future studies.

There is evidence to suggest (albeit from small studies) that an infected and inflamed periodontium is associated with increased severity of OM [24•, 38–41, 81]. Well-powered prospective observational studies in homogenous groups of patients, using well-defined parameters for the assessment of OM and periodontal disease, are necessary to substantiate this association.

Interestingly, it has been proposed that the presence of inflammation (anywhere in the body) primes for a dysregulated and exaggerated inflammatory response following a subsequent inflammatory stimulus [82]. This “two-hit model” has been hypothesized to underpin an association between periodontitis and OM [83•]. The authors postulated that pre-existing periodontitis may co-induce an exaggerated inflammatory response following radiotherapy in patients with HNC, leading to more severe OM. In turn, OM may contribute to the severity of periodontitis. Taking this hypothesis one step further, it seems feasible that not only may periodontitis serve as a “first hit” for mucositis, but that any oral or non-oral inflammatory-driven toxicity may co-induce other inflammatory complications (e.g., cachexia, fatigue, systemic inflammatory response syndrome). This theory may also explain the well-documented observation that cancer regimen-related toxicities do not occur in isolation, but rather develop in non-random clusters [55, 84, 85]. Moreover, a patient’s risk of developing both periodontitis and inflammatory-driven toxicities may be linked by a genetic predisposition to express increased levels of inflammatory mediators. These intriguing hypotheses deserve further exploration.

In addition, investigations should be performed to further characterize the role of the oral environment. This includes studies on the potential contribution of the oral/periodontal microbiome in the pathobiology of OM and stomatitis associated with targeted therapies using next-generation sequencing techniques. Similarly, studies on changes in salivary output and salivary proteome induced by cancer therapies may contribute to a scientific base for OM risk prediction, early diagnosis, and interventions.

Furthermore, there is a pressing need for clinical practice guidelines for periodontal management in cancer patients, from diagnosis through survivorship. In particular, strategies are needed for effectively minimizing periodontal infection and inflammation in these often medically compromised patients. In addition to debridement, approaches directed toward reducing periodontal inflammation seem to hold promise [86]. Among other strategies, adjunctive host modulation therapy developed for periodontitis (i.e., sub-antimicrobial-dose doxycycline alone or in combination with an anti-inflammatory agent) may simultaneously mitigate both periodontitis and other inflammatory conditions [87••]. These interventions may open new avenues for the management of periodontitis, but may also positively affect OM and other inflammatory complications associated with cytotoxic cancer therapies.

## Conclusions

Oral mucosal toxicities associated with antineoplastic therapies continue to represent a significant oncological challenge. While our understanding of oral adverse effects associated with targeted therapies is still in its infancy, and knowledge of the epidemiology of OM is incomplete, significant progress has been made in unraveling the pathogenesis of OM, and some preventive approaches have been identified. Nevertheless, for the majority of patients, no effective interventions are available.

Researchers and clinicians in the field of cancer regimen-related toxicities and those involved in oral and periodontal medicine should join forces in pursuit of understanding and developing strategies for treatment of inflammatory conditions in oncology. Ultimately, this will lead to effective interventions and will reduce the burden of OM and other toxicities associated with cancer treatment.
